# INDICATION FOR LIVER TRANSPLANTATION DUE TO HEPATOCELLULAR CARCINOMA: ANALYSIS OF 1,706 PROCEDURES OVER THE PAST DECADE IN THE STATE OF PARANÁ

**DOI:** 10.1590/0102-672020220002e1701

**Published:** 2022-12-19

**Authors:** Alexandre Coutinho Teixeira de Freitas, Fátima Diana Samúdio Espinoza, Cristina Alvarez Mattar, Júlio Cezar Uili Coelho

**Affiliations:** 1 Universidade Federal do Paraná, Department of Surgery and Liver Transplant Unit – Curitiba (PR), Brazil.

**Keywords:** Liver Transplantation, Carcinoma, Hepatocellular, Waiting Lists, Transplante de Fígado, Carcinoma Hepatocelular, Listas de Espera

## Abstract

**BACKGROUND:**

Patients listed for liver transplantation and hepatocellular carcinoma are considered priority on the waiting list, and this could overly favor them.

**AIM:**

This study aimed to evaluate the impact of this prioritization.

**METHODS:**

We analyzed the liver transplants performed in adults from 2011 to 2020 and divided into three groups: adjusted Model of End-Stage Liver Disease (MELD) score for hepatocellular carcinoma, other adjusted Model of End-Stage Liver Disease situations, and no adjusted Model of End-Stage Liver Disease.

**RESULTS:**

A total of 1,706 patients were included in the study, of which 70.2% were male. Alcoholism was the main etiology of cirrhosis (29.6%). Of the total, 305 patients were with hepatocellular carcinoma, 86 with other adjusted Model of End-Stage Liver Disease situations, and 1,315 with no adjusted Model of End-Stage Liver Disease. Patients with hepatocellular carcinoma were older (58.9 vs. 53.5 years). The predominant etiology of cirrhosis was viral hepatitis (60%). The findings showed that group with adjusted Model of End-Stage Liver Disease had lower physiological Model of End-Stage Liver Disease (10.9), higher adjusted Model of End-Stage Liver Disease (22.6), and longer waiting list time (131 vs. 110 days), as compared to the group with no adjusted Model of End-Stage Liver Disease. The total number of transplants and the proportion of patients transplanted for hepatocellular carcinoma increased from 2011 to 2020. There was a reduction in the proportion of patients with hepatocellular carcinoma and adjusted Model of End-Stage Liver Disease of 20 and there was an increase on waiting list time in this group. There was an increase in the proportion of those with adjusted Model of End-Stage Liver Disease of 24 and 29, but the waiting list time remained stable.

**CONCLUSION:**

Over the past decade, prioritization of hepatocellular carcinoma resulted in an increased proportion of transplanted patients in relation to those with no priority. It also increased waiting list time, requiring higher adjusted Model of End-Stage Liver Disease to transplant an organ.

## RESUMO

**RACIONAL:**

Pacientes portadores de carcinoma hepatocelular com indicação de transplante hepático recebem prioridade na lista de espera e isso poderia favorecê-los demasiadamente.

**OBJETIVO:**

Avaliar o impacto dessa priorização.

**MÉTODOS:**

Foram analisados os transplantes hepáticos realizados de 2011 até 2020 no estado do Paraná, divididos em três grupos: portadores de carcinoma hepatocelular no modelo para doença hepática terminal (MELD) ajustado, outras situações no modelo para doença hepática terminal ajustado e sem o modelo para doença hepática terminal ajustado.

**RESULTADOS:**

Foram incluídos 1.706 pacientes, 70,2% do gênero masculino, a maioria portadores de cirrose alcoólica (29,6%): 305 com hepatocarcinoma, 86 com outras situações no modelo para doença hepática terminal ajustado e 1.315 sem o modelo para doença hepática terminal ajustado. Nos portadores de hepatocarcinoma, a idade média foi maior (58,9 *vs* 53,5 anos), a etiologia predominante da cirrose foram as hepatites virais (60%), apresentaram menor no modelo para doença hepática terminal fisiológico (10,9), maior no modelo para doença hepática terminal corrigido (22,6 *vs* 21,8) e maior tempo em lista de espera (131 *vs* 110 dias) quando comparados ao grupo sem o modelo para doença hepática terminal ajustado. O número de transplantes e a proporção de pacientes transplantados por hepatocarcinoma aumentou de 2011 até 2020. Houve redução da proporção dos portadores de hepatocarcinoma com o modelo para doença hepática terminal de 20 no decorrer da década e aumento do tempo em lista nesse grupo. Para os com modelo para doença hepática terminalde 24 e de 29, houve aumento na proporção e o tempo em lista permaneceu estável.

**CONCLUSÃO:**

A priorização do hepatocarcinoma conferiu maior modelo para doença hepática terminal e incremento na proporção de transplantes em relação aos sem prioridade. Também aumentou o tempo em lista de espera, necessitando maior modelo para doença hepática terminal corrigido para obtenção de um órgão.

## INTRODUCTION

Liver transplantation is the current treatment of choice for patients with end-stage liver disease. However, the number of organs available does not follow the growth of the number of patients on the waiting list for transplantation^12^. In Brazil, to reduce the mortality of patients on the waiting list and optimize the distribution of organs, the Ministry of Health adopted the Model of End-Stage Liver Disease (MELD) as criteria to classify patients according to the severity of cirrhosis^4,7^.

Hepatocellular carcinoma, the most prevalent malignant neoplasm of the liver in the world, is related to chronic liver diseases^17^. It is a condition in which the definitive treatment is liver transplantation, as it cures both cirrhosis and cancer^17,18^. However, the prognosis of this disease is mainly influenced by tumor progression and not by the parenchymal disease. MELD score is frequently low in this situation, thus reducing the chance to transplant an organ^9^. Therefore, in order to balance this chance, in Brazil, there is a specific policy to hepatocellular carcinoma. Patients within the Milan criteria receive an adjusted MELD score of 20, regardless of its physiological value^11^. If transplant is not performed within 3 months, the adjusted MELD score automatically changes to 24; and, in 6 months, to 29. This is done to reduce waiting list time and avoid progression of the disease beyond Milan criteria, leaving no other effective alternative treatment^10^.

Some authors claim that patients with hepatocellular carcinoma would be excessively favored with this policy^16,19^. For this reason, some changes have been implemented in some countries. In the United States, patients with hepatocellular carcinoma have their MELD score adjusted only after 6 months on the waiting list^14,19^. There is a lack of data about this subject in Brazil.

This study aimed to evaluate the impact of the Brazilian policy for patients with hepatocellular carcinoma in the indication of liver transplants performed in the State of Paraná over the past decade.

## METHODS

The following data were collected at Parana's State Transplant Agency database: patient's name, date of birth, gender, date of inclusion on the transplant list, date of transplantation, etiology of cirrhosis, MELD, and adjusted MELD. The inclusion criteria were patients over 18 years of age subjected to liver transplantation in the State of Paraná from January 2011 to December 2020. Pediatric transplants or patients with incomplete data were excluded.

Patients were divided into three groups:

1. Patients with hepatocellular carcinoma with adjusted MELD score
2. Patients with other clinical situations in which Brazilian legislation allows to adjust the MELD score
3. Patients without adjusted MELD score

Patients with hepatocellular carcinoma and adjusted MELD score were also divided into three subgroups according to the score at the time of transplantation: MELD of 20, MELD of 24, and MELD of 29.

Groups were compared according to gender, age, etiology of cirrhosis, MELD score, and time on the waiting list. In addition, an evolutionary comparison was made between 2011 and 2020 of the following data: absolute number and percentage of transplants, MELD, and time on the waiting list among the three main groups and among the three subgroups of patients with hepatocellular carcinoma.

For the association between the study data, the Mann-Whitney, Kruskal-Wallis, and chi-square tests were performed. The level of statistical significance was set at 5% (p=0.05). The Jamovi Project (2020) version 1.6 statistical software was used.

The study was approved by the Federal University of Paraná Health Sciences Sector Ethical Committee, approval number 42264521.5.0000.0102, with agreement of Paraná State Transplant Agency.

## RESULTS

From January 2011 to December 2020, 1,785 liver transplants were initially selected and 1,706 were included in the study. Reasons for exclusion were age under 18 years (n=67) and incomplete data in the Transplant Registry (n=12).

The characteristics of the recipients are shown in Table 1. Of the 1,706 recipients, 305 had adjusted MELD score for hepatocellular carcinoma, 86 had adjusted MELD score for other situations, and 1,315 had no adjusted MELD score. The proportion of women was superior to men only in the group of adjusted MELD score for other situations (51.2% vs. 48.8%; p<0.001). Patients with hepatocellular carcinoma were older (58.9±8.7 years; p<0.001).

**Table 1:** General characteristics.

		Adjusted MELD score for HCC	Other adjusted MELD score situations	No adjusted MELD score	Total	p-value
n (%)		305 (17.9)	86 (5.0)	1,315 (77.1)	1,706 (100)	
Gender, n (%)						
	Male	234 (76.7)	42 (48.8)	921 (70.0)	1,197 (70.2)	<0.001*
	Female	71 (23.3)	44 (51.2)	394 (30.0)	509 (29.8)	
Age (years)		58.9 ± 8.7	48 ± 12.9	52.6±11.3	53.5±11.3	<0.001*
Etiology of cirrhosis, n (%)						
	Alcohol	52 (17.0)	8 (9.3)	445 (33.8)	505 (29.6)	<0.001*
	Viral hepatitis	183 (60.0)	8 (9.3)	292 (22.2)	483 (28.3)	<0.001*
	Cryptogenic	14 (4.6)	2 (2.3)	188 (14.3)	204 (12.0)	<0.001*
	Other	23 (7.5)	56 (65.1)	114 (8.7)	193 (11.3)	<0.001*
	NASH	24 (7.9)	2 (2.3)	117 (8.9)	143 (8.4)	0.097*
	Autoimmune hepatitis	3 (1.0)	3 (3.5)	69 (5.2)	75 (4.4)	0.004*
	Primary biliary cirrhosis	4 (1.3)	6 (7.0)	29 (2.2)	39 (2.3)	0.007*
	Fulminant hepatitis	1 (0.3)	0 (0.0)	35 (2.7)	36 (2.1)	0.014*
	Secondary biliary cirrhosis	1 (0.3)	1 (1.2)	26 (2.0)	28 (1.6)	0.116*
MELD		10.9	15.4	21.8	19.5	<0.001*
Adjusted MELD score		22.6	26.9	21.8	23.5	<0.001*
Waiting time (days)		131.9	131.4	110.6	115.4	<0.001*

* Chi-square;

** Kruskal-Wallis; HCC: hepatocellular carcinoma; NASH: nonalcoholic steatohepatitis; MELD: Model of End-Stage Liver Disease.

The main etiology of cirrhosis was alcoholism, both in patients with no adjusted MELD score (29.6%) and in the group with adjusted MELD score for other situations (33.8%). In the group of patients with hepatocellular carcinoma, viral hepatitis represented the main cause of cirrhosis (60%; n=183), the cause was alcoholic in only 17% (n=52).

Table 2 shows the annual number of liver transplants performed for each group. Over the decade, there was a significant increase in the number of transplants performed in the State of Paraná: there were 47 transplants in 2011 and 222 transplants in 2020. The absolute number and proportion of transplants for hepatocellular carcinoma in relation to patients with no adjusted MELD score increased from 2011 to 2020 (p<0.001).

**Table 2:** Number of liver transplantations on each group.

Year	Adjusted MELD score for HCC* n (%)	Other adjusted MELD score situations n (%)	No adjusted MELD scores n (%)	Total	p-value
2011	10 (21.3)	0 (0.0)	37 (78.7)	47	<0.001*
2012	19 (20.6)	2 (2.2)	71 (77.2)	92	
2013	27 (26.7)	5 (5.0)	69 (68.3)	101	
2014	28 (31.1)	6 (6.7)	56 (62.2)	90	
2015	36 (28.6)	10 (7.9)	80 (63.5)	126	
2016	31 (14.8)	13 (6.2)	166 (79.0)	210	
2017	32 (12.1)	3 (1.1)	230 (86.8)	265	
2018	45 (14.9)	18 (6.0)	238 (79.1)	301	
2019	35 (13.9)	10 (4.0)	207 (82.1)	252	
2020	42 (18.9)	19 (8.6)	161 (72.5)	222	
Total	305	86	1,315	1,706	

* Chi-square; HCC: hepatocellular carcinoma; MELD: Model of End-Stage Liver Disease.

Table 3 compares the MELD scores within the groups. The group with adjusted MELD score for other situations had the highest values over the years (p<0.001). The hepatocellular carcinoma group had higher adjusted and lower physiological MELD scores over the years when compared to the group with no adjusted MELD score (p<0.001). The years 2016 and 2017 were an exception in relation to the adjusted MELD score. When the whole decade was considered, the group with hepatocellular carcinoma had lower physiologic and higher adjusted MELD score than the group with no adjusted MELD score (10.9 and 22.6 vs. 21.8; p<0.001) and lower adjusted and physiologic MELD score than the group with adjusted MELD score for other situations (22.6 and 10.9 vs. 26.9 and 15.4; p<0.001).

**Table 3:** Model of End-Stage Liver Disease scores comparison over the years.

Year	Adjusted MELD score for HCC		Other adjusted MELD score situations		No adjusted MELD score	p-value
	MELD	Adjusted MELD	MELD	Adjusted MELD	MELD	
2011	11.5	20.4	–	–	19.4	<0.001*
2012	11.1	20.8	17.0	40.0	20.9	<0.001*
2013	10.6	22.1	16.8	28.8	21.2	<0.001*
2014	10.8	24.0	24.3	34.3	23.6	<0.001*
2015	12.1	22.9	10.6	28.4	21.3	<0.001*
2016	9.9	21.7	10.4	24.4	22.4	<0.001*
2017	10.8	21.2	23.0	27.0	23.5	<0.001*
2018	10.8	21.9	11.9	22.8	21.0	<0.001*
2019	11.3	24.5	19.8	27.6	21.0	<0.001*
2020	10.9	23.8	18.0	26.9	21.6	<0.001*
Total	10.9	22.6	15.4	26.9	21.8	<0.001*
p-value	0.379*	<0.001*	0.005*	0.032*	<0.001*	–

* Kruskal-Wallis; HCC: hepatocellular carcinoma; MELD: Model of End-Stage Liver Disease.

The number of transplants performed annually in patients with hepatocellular carcinoma and classified according to the adjusted MELD score is shown in Table 4. It was observed that the number of transplants performed with adjusted MELD score of 29 increased over the years (p<0.001): in 2014, they were only 13.9% (n=5) of the transplants performed; in 2020, they were only 30.6% (n=11). The proportion of transplants with adjusted MELD score of 24 also increased. In contrast, the proportion of transplants with MELD score of 20 decreased (p<0.001).

**Table 4:** Annual transplants according to adjusted Model of End-Stage Liver Disease score in patients with hepatocellular carcinoma.

Year	Adjusted MELD score for HCC			p-value
	20	24	29	
	n (%)	n (%)	n (%)	
2011	9 (5.9)	1 (0.9)	0 (0.0)	<0.001*
2012	15 (9.8)	4 (3.4)	0 (0.0)	
2013	13 (8.5)	14 (12.1)	0 (0.0)	
2014	6 (3.9)	17 (14.7)	5 (13.9)	
2015	16 (10.5)	15 (12.9)	5 (13.9)	
2016	20 (13.1)	9 (7.8)	2 (5.6)	
2017	24 (15.7)	7 (6.0)	1 (2.8)	
2018	25 (16.3)	19 (16.4)	1 (2.8)	
2019	9 (5.9)	15 (12.9)	11 (30.6)	
2020	16 (10.5)	15 (12.9)	11 (30.6)	
Total	153 (100)	116 (100)	36 (100)	

* Chi-square; HCC: hepatocellular carcinoma; MELD: Model of End-Stage Liver Disease.

Table 5 shows time on the waiting list for each group. It was similar from 2011 to 2017 when compared the three groups year by year. In 2018, the group with adjusted MELD score for other situations had longer waiting time. In 2019 and 2020, the waiting time was longer in patients with hepatocellular carcinoma when compared with the group with no adjusted MELD score (167.3 and 207.1 days vs. 91.1 and 132.5 days; p<0.001). When the whole decade was considered, the group with hepatocellular carcinoma has longer waiting time than the group with no adjusted MELD score (131.9 vs. 110.6 days; p<0.001) and similar waiting time than the group with adjusted MELD score for other situations (131.4; p=0.415).

**Table 5:** Time on waiting list for liver transplantation.

Year	Waiting time (days)			Total	p-value
	Adjusted MELD score for HCC	Other adjusted MELD score situations	No adjusted MELD score		
2011	267.0	–	298.1	291.4	0.917**
2012	72.2	67.5	138.4	123.2	0.171*
2013	105.2	101.2	116.9	113.0	0.404*
2014	146.3	53.9	132.9	131.9	0.059*
2015	146.3	260.6	146.1	155.2	0.101*
2016	87.3	156.6	102.0	103.2	0.033*
2017	70.9	64.3	69.7	69.8	0.002*
2018	98.8	127.1	101.9	102.9	<0.001*
2019	167.3	88.7	91.1	101.6	<0.001*
2020	207.1	122.3	132.5	145.7	<0.001*
Total	131.9	131.4	110.6	115.4	<0.001
p-value	<0.001*	0.415*	<0.001*	<0.001*	–

* Kruskal-Wallis;

** Mann-Whitney; HCC: hepatocellular carcinoma; MELD: Model of End-Stage Liver Disease.

Time on the waiting list for patients with hepatocellular carcinoma and classified according to the adjusted MELD score subdivision is shown in Table 6. For the group with adjusted MELD score of 20, there was an increase on waiting list time over the decade; for the groups with adjusted MELD score of 24 and 29, waiting list time remained stable, except in 2017, when the time was shorter for both. Time on waiting list was higher on patients with adjusted MELD score of 29 in relation to patients with adjusted MELD score of 24 and 20. It was also higher in patients with adjusted MELD of 24 in relation to patients with adjusted MELD score of 20.

**Table 6:** Time on waiting list for patients with hepatocellular carcinoma according to adjusted Model of End-Stage Liver Disease score subdivision.

Year	Waiting time (days)			p-value
	Adjusted MELD of 20	Adjusted MELD of 24	Adjusted MELD of 29	
2011	278.9	160.0	–	0.8**
2012	54.9	137.0	–	0.147**
2013	83.8	125.0	–	0.008**
2014	63.3	129.8	304.2	<0.001*
2015	67.3	139.0	421.4	<0.001*
2016	47.2	137.1	310	<0.001*
2017	72.8	66.4	57.0	0.997*
2018	55.2	151.1	196.0	<0.001*
2019	75.0	187.7	214.6	<0.001*
2020	147.6	182.4	327.5	<0.001*
p-value	0.006*	0.036*	0.035*	–
Total	84.9	144.7	290.7	<0.001*

* Kruskal-Wallis;

** Mann-Whitney; MELD: Model of End-Stage Liver Disease.

## DISCUSSION

The total number of liver transplants performed in the State of Paraná has increased significantly over the past decade. In absolute liver transplants number per year, Brazil is ranked in second place in the world, behind the United States. Paraná ranks in second place among all Brazilian states when analyzing the number per million inhabitants^2^. This study also showed that the absolute number and the proportion of liver transplants for patients with hepatocellular carcinoma and adjusted MELD score have increased since 2011 when compared to those without adjusted MELD. This corroborates the findings of other studies, and the implantation of MELD score as the waiting list criteria is stated as the cause^3,19,21^. This increase was also demonstrated in liver transplants done in the state of Rio Grande do Sul^1,16^.

Viral hepatitis was the main cause of cirrhosis in patients with hepatocellular carcinoma, accounting for 60% of cases. This is explained by carcinogenic factors of chronic infection by hepatitis B and C viruses that lead to fibrosis and liver cirrhosis^8^. In this group, patients were older when compared to patients with no adjusted MELD score. These findings corroborate those of Carrillo et al. and Schlansky et al.^5,18^.

MELD has revolutionized the waiting list classifying method for liver transplantation^15^. However, the minimum score needed to receive an organ is increasingly higher, mainly due to the adoption of exception points, as in the case of hepatocellular carcinoma^13^. Rodriguez et al. analyzed liver transplants performed for hepatocellular carcinoma at a reference center in Porto Alegre (Brazil) between 2007 and 2016^16^. Their physiological MELD score was lower compared to patients with no adjusted MELD (11.8 vs. 18.19), result consistent with this study and a North American study^13^. Those findings show that Brazilian legislation giving privilege to transplants for hepatocellular carcinoma is very effective, as the minimum MELD score for these patients is 20. In Singapore, patients with hepatocellular carcinoma immediately receive an adjusted MELD score of 15, with no increase over time^20^. The study found that this policy do not favor patients with hepatocellular carcinoma as this MELD score is lower than the value in patients with no adjusted MELD score (15 vs. 20).

Our study also showed an increase in the proportion of transplants for hepatocellular carcinoma with adjusted MELD score of 24 and 29. The reason is that as more patients are listed with exception MELD scores, they compete with each other for an organ and spend more time on the waiting list.

If we consider the past decade, time on waiting list was longer in patients with hepatocellular carcinoma in relation to patients with no adjusted MELD score. This happened because the increased waiting time observed in 2019 and 2020. Until this period, waiting time was similar between groups. As mentioned previously, higher adjusted MELD scores were necessary and, according to the Brazilian legislation, patients migrate from one adjusted MELD score subcategory to another only after 3 months waiting time. Other authors found different results. In the study by Rodriguez et al., patients listed for hepatocellular carcinoma underwent transplantation after 5.6 months, while in patients without hepatocarcinoma it was after 25 months^16^. Bhat et al. found no difference on waiting time for patients with hepatocellular carcinoma^3^.

Just after the implementation of MELD in the United States, the waiting list time for liver transplantation fell from 2.28 to 0.69 years in patients with hepatocellular carcinoma^19^. After 3 months, 87% of patients with hepatocellular carcinoma underwent liver transplantation. The conclusion was that excessive prioritization was occurring to these patients. In 2015, exception points policy was changed in the United States. Since then, patients are listed initially with their physiological MELD score, and only after 6 months they receive an adjusted score of 28^14,16,19^. Brazilian policy is very similar in some aspects. The maximum adjusted MELD score is 29 and it is obtained only after 6 months in the waiting list. The main difference is that these patients progressively obtain that score. They are initially listed with adjusted MELD score of 20 and, if not transplanted after 3 months, it progresses to 24 and again to 29 after 3 more months waiting. As discussed previously, our results showed historical similarity in terms of waiting list time between the groups. More recently, patients with hepatocellular carcinoma had even increased waiting time for an organ. It seems Brazilian policy is not excessively favoring these patients.

The worldwide shortage of organs for transplantation demands for inclusion criteria on the waiting list. Patients with hepatocellular carcinoma can be removed from the list if tumor progression occurs. The exclusion rate is 7–11% in 6 months and up to 40% in 1 year^22^. Although increased waiting time may remove some patients from the list, only those who have tumors with more favorable prognosis are selected. This positively influences post-transplant survival rates^6^. The opposite occurs with patients without hepatocellular carcinoma. Longer waiting time for an organ causes lower survival rates^18^.

The Milan criteria are the most used to define which patients with hepatocellular carcinoma are eligible for liver transplantation. However, some authors believe that it is too restrictive^23^. For this reason, other criteria, such as the University of California, have been suggested^24^. It assumes that a modest expansion of Milan criteria does not negatively affect post-transplant survival and it is even better for predicting prognosis^23,24^.

The purpose of adjusted MELD score is to ensure access to liver transplantation for all groups of patients. This seems to occur in the State of Paraná, although data about the number of patients excluded from the waiting list due to tumor progression beyond Milan criteria are still lacking. Although the proportion of patients transplanted for hepatocellular carcinoma has increased in the past decade, it seems that this process is now stabilized. Transplanted patients with no adjusted MELD score are still the vast majority.

## CONCLUSION

The number of liver transplants in the State of Paraná increased over the past decade. This increment is proportionally higher in patients with hepatocellular carcinoma because of the Brazilian policy to increase the MELD of this group of patients. Nonetheless, it was progressively necessary higher adjusted MELD scores and longer time on waiting list to succeed that.
